# Towards Realistic Urban Traffic Experiments Using DFROUTER: Heuristic, Validation and Extensions

**DOI:** 10.3390/s17122921

**Published:** 2017-12-15

**Authors:** Jorge Luis Zambrano-Martinez, Carlos T. Calafate, David Soler, Juan-Carlos Cano

**Affiliations:** 1 Department of Computer Engineering (DISCA), Universitat Politècnica de València, 46022 Valencia, Spain; calafate@disca.upv.es (C.T.C.); jucano@disca.upv.es (J.-C.C.); 2 Institute of Multidisciplinary Mathematics (IMM), Universitat Politècnica de València, 46022 Valencia, Spain; dsoler@mat.upv.es

**Keywords:** SUMO, DFROUTER, heuristic, O-D matrix, intelligent transportation system, vehicles, detectors, computational modeling, mathematical model

## Abstract

Traffic congestion is an important problem faced by Intelligent Transportation Systems (ITS), requiring models that allow predicting the impact of different solutions on urban traffic flow. Such an approach typically requires the use of simulations, which should be as realistic as possible. However, achieving high degrees of realism can be complex when the actual traffic patterns, defined through an Origin/Destination (O-D) matrix for the vehicles in a city, remain unknown. Thus, the main contribution of this paper is a heuristic for improving traffic congestion modeling. In particular, we propose a procedure that, starting from real induction loop measurements made available by traffic authorities, iteratively refines the output of DFROUTER, which is a module provided by the SUMO (Simulation of Urban MObility) tool. This way, it is able to generate an O-D matrix for traffic that resembles the real traffic distribution and that can be directly imported by SUMO. We apply our technique to the city of Valencia, and we then compare the obtained results against other existing traffic mobility data for the cities of Cologne (Germany) and Bologna (Italy), thereby validating our approach. We also use our technique to determine what degree of congestion is expectable if certain conditions cause additional traffic to circulate in the city, adopting both a uniform pattern and a hotspot-based pattern for traffic injection to demonstrate how to regulate the overall number of vehicles in the city. This study allows evaluating the impact of vehicle flow changes on the overall traffic congestion levels.

## 1. Introduction

In urban areas, high population densities are related to traffic problems such as CO${}_{2}$ emissions, accidents, noise and environmental pollution, all of them being critical issues for city authorities. Concerning solutions to reduce the amount of traffic, or to improve the traffic flow in a city, several alternatives have been proposed such as using public transportation, or limiting the access of vehicles to downtown areas depending on their license plate number. In addition, a detailed traffic analysis is often necessary to find ways to improve traffic flow. Traffic management solutions typically require the use of simulators able to capture in detail all the particular characteristics and dependencies associated with real-life traffic. These simulations depend on different factors such as vehicle speed, vehicle density, traffic flow and the environment itself.

Mobility models can be classified according to the level of detail to represent the traffic system as four types: macroscopic, mesoscopic, microscopic and sub-microscopic [1]. Macroscopic models simplify the analysis by describing traffic at high levels of aggregation. This model assumes that traffic is properly allocated to the roadway lanes, and individual vehicle manoeuvres are not explicitly represented. Therefore, their computational requirements are low, but the results provided have little accuracy and representativeness. Mesoscopic models represent transportation systems by analyzing the behavior of drivers. The do not distinguish the traces of individual vehicles, rather specifying the behavior of individuals, based on which the probability functions required for a vehicle to move at a certain speed are defined, as well as the probability of being located in a particular position at a specific time. Traffic is represented by small groups of traffic entities, activities and iterations, thus introducing a moderate level of detail. An instantaneous event (change of maneuvering lane) is represented by an individual vehicle, where the decision to perform this action is based on differences in speed and relative lane densities. Some models are derived in analogy to gas-kinetic theory, which describes the dynamics of velocity distribution. Microscopic models describe the time-space behavior of the traffic (vehicles and drivers), as well as their interaction, individually, thereby achieving a high level of detail. Thus, microscopic models are able to represent real situations with a higher accuracy since each vehicle becomes an independent node in the simulation. Finally, sub-microscopic models describe the individual characteristics of the vehicles in the traffic, detailing the driving behavior, as well as the control of the vehicle (e.g., changing gears, autonomous intelligent cruise control operations, etc.), in correspondence with the various conditions that are modeled in detail. In addition, the operations of specific parts or sub-units of the vehicle are described, significantly increasing computational complexity.

According to Härri et al. [2], traffic models can be also classified as: (i) synthetic models, which include all mathematical models such as stochastic, traffic stream and queue models; (ii) survey-based models, which extract mobility patterns from surveys and are mostly applicable to macroscopic models; (iii) trace-based models, where mobility patterns are obtained from real mobility traces; these are the most useful, but their availability is limited; and finally, (iv) traffic simulator-based models, where a traffic simulator such as SUMO (Simulation of Urban MObility) [3] is responsible for managing the actual vehicular mobility based on either synthetic or realistic traces.

For vehicular studies to be meaningful, it is important to count on representative and accurate traffic models (traffic simulators), which usually requires knowing the origin and destination of every trip (represented as an origin/destination matrix). However, obtaining such matrices is not straightforward, and many traffic administration authorities try to find new ways to obtain this information.

In this work, we extend our previous conference contribution [4] by proposing a procedure that allows creating scenarios based on realistic mobility models that are useful when simulating large-scale traffic in urban areas. We will focus on the city of Valencia, Spain, where real traffic data are introduced in the SUMO mobility simulation tool. In particular, we start from induction loop measurements made available by the City Hall of Valencia (see [5] for more details), and we then propose a heuristic that iteratively refines the output produced by the DFROUTER tool [6] which consists of a list of routes and a list of vehicles associated with each route. This allows determining where vehicles are coming from and what are their destinations. Those vehicles will be injected into the simulation network at their respective positions, thereby resembling the real traffic distribution. Notice that the DFROUTER tool works similarly to a static O-D matrix estimation, but it does not work as an O-D estimator, so it cannot be compared with O-D matrix estimation methods. For this reason, SUMO performs special calculations in the simulation to obtain more parameters for the traffic [7].

To validate the results obtained, we then compared them through simulation against other existing traffic mobility data for the cities of Cologne and Bologna. Additionally, since traffic planning and optimization typically require studying the impact of unexpected traffic load conditions, we also detail how, based on our reference scenario, we can regulate the amount of vehicles in the city to generate different degrees of congestion. This allows determining which degree of congestion is expectable in different situations; in particular, we study the impact of having an additional traffic load on the overall traffic congestion levels when adopting either a uniform or a hotspot-based pattern for traffic injection.

The paper is organized as follows: in Section 2, we present some related works regarding the process of generating realistic mobility models. Section 3 provides details about the tools used in our work i.e., SUMO and DFROUTER. Section 4 describes the proposed technique, which is based on complementing DFROUTER in order to adjust the number of vehicles injected into the network to mimic real traces. The results achieved, and a comparative analysis of two previous models using data from the cities of Bologna and Cologne, are then presented in Section 5. The impact of varying the degree of traffic congestion is also studied in Section 6, where both a uniform load model and a hotspot-based model are proposed and evaluated. Finally, Section 7 concludes the paper.

## 2. Related Works

In the last few decades, ITS have experienced an outstanding growth, being considered as the most efficient way to improve the performance of urban traffic flows.

Some positioning technologies such as the Global Navigation Satellite System (GNSS), alongside an increasing popularity of cell phones, have emerged to monitor traffic conditions in real time [8], providing realistic travel information and allowing one to estimate travel demand. Ma et al. [9] propose to predict the evolution of large-scale traffic congestion based on data from the Global Positioning System, using a deep restricted Boltzmann machine and recurrent neural network architecture.

Different methods have been proposed for obtaining the O-D matrix. Cascetta [10] proposes the combination of direct estimators or models with traffic counts via an assignment model with a generalized least squares estimator of the O-D matrix. Caceres et al. [11] propose a method for storing location update events to measure the flow of O-D trips between each location area by transforming cellular phone counts into vehicle counts. Sohn and Kim [12] develop an idle handoff technology for positioning cellular phones to get a virtual traffic count, and then, they used synthetic methods to derive a time-dependent O-D matrix based on traffic counts. Pan et al. [13] propose recording the position of cellular phones every two hours to obtain the trip distribution between each set of O-D elements. Concerning these previous works, we have found two limitations. Firstly, as the “location area” includes dozens of cells and since their coverage is much larger than a cell, merging handoffs and location updates increases the complexity when estimating O-D matrix values. Secondly, converting cellular phone counts into vehicle counts can lead to inaccurate positions. Notice that cellular phone counts are not equal to vehicle counts because nowadays, vehicles tend to carry several cell phones simultaneously. Another method to obtain the O-D matrix is through mathematical equations to obtain the possible estimates. According to Wang et al. [14], a two-stage algorithm is proposed to simultaneously estimate the O-D matrix and the link choice proportion in a congested network using a dispersion parameter for partial traffic counts. Castillo et al. [15] determined which is the subset of variables that can be calculated with another subset of variables that have been observed, thereby solving the traffic network observability problem. Later, Castillo et al. [16] proposed a mathematical theorem that defines the sufficient conditions and methods required to solve the observability problems in traffic networks.

Bluetooth technology has also been used as a sensor for measuring traffic since 2005. Vehicles carrying detectable Bluetooth devices can be detected by Bluetooth sensors, which are installed at multiple locations along the roads. These sensors record the Bluetooth MAC address and the time it was detected, which can be used for travel time estimation [17]. A system based on a wireless sensor network is presented by Fernández-Lozano et al. [18], and it obtains traffic information to characterize urban traffic, particularly by monitoring it through the calculation of the origin-destination matrix using Bluetooth identification. According to Lees-Miller et al. [19], the main challenges of this technology are position accuracy and missed detections. Notice that, although the detection time is known precisely, the position of a detected vehicle is not precise, meaning that the device can be anywhere within the detection range. Increasing this accuracy requires reducing the detection range by tuning the antenna characteristics and the transmission power level. In addition, it is frequent that a device passes through a detector without being detected. This is caused by the random delay in the process of device detection defined for Bluetooth, which can be up to 10 s even under ideal radio conditions [20]. This means that, if we consider a maximum radius of 10 m, a vehicle to be detected should have a speed of 2m/s (7.2km/h), which is not realistic.

When focusing instead on traffic flow monitoring and obtaining O-D matrices, special detectors can be deployed through a vehicular network, such as induction loops. The data supplied by those devices are vehicle presence, count and occupancy. The induction loops may have poor reliability, especially when the connections are incorrect and even when installed on defective pavement or in areas where utilities frequently dig up the roadbed. Another disadvantage that can be presented is not having a direct measurement of the speed of vehicles. Zhu et al. [21] propose a network sensor location problem model that integrates the deployment of heterogeneous sensors in the most optimal way, including link sensors such as induction loop detectors and node sensors such as camera or video, thereby maximizing the quality of the O-D matrix estimation under a budget constraint. In this study, induction loops are used for the counting of vehicles in the lanes or set of lanes in the vehicular network, and the node sensors are installed at the intersections to detect the turning movements. Zijpp [22] proposed a method to track time-varying traffic patterns by combining link volume counts and trajectory observations. This method can be applied if accurate O-D travel information is available. Barceló Bugeda et al. [23] focus on the estimation of time-dependent O-D matrices from measurements of traffic variables. They assume that the usual traffic data collected by inductive loop detectors are complemented by accurate measurements of travel times and speeds between two consecutive sensors, such as using Bluetooth devices on board vehicles. Yang et al. [24] proposed two origin-destination flow estimation models using link flow counts and the GPS positions of probe vehicles. The first model uses a scaled probe vehicle O-D matrix as a preliminary O-D matrix and then applies a generalized least square framework for O-D correction using link counts. The second model is an extension of the first model in which the observed link probe ratios are included as additional information in the O-D estimation process. On the other hand, Xie et al. [25] focuses on the street intersection, which is independently controlled by an on-line scheduling agent whose objective is to minimize the total accumulated delay of vehicles traveling through the road network over a period of time and optimize traffic flow using a schedule to determine the best control action at intersections. Likewise, McCluskey et al. [26] propose to investigate the application of linked data in order to improve the source of environmental and traffic data, and they use automated planning tools to allow traffic to be managed, thereby avoiding air pollution problems, focusing on the planning of the phases of traffic lights and deciding the best flow of traffic prioritized in terms of vehicle numbers in some critical road links.

Finally, a great number of ITS applications are implemented with vision-driven technologies that collect data from video-based detection like vehicle density, traffic behavior analysis and statistical traffic data analysis. However, these techniques also have their limitations and drawbacks. Their main challenging issues are: (i) the wide variety of vehicles in shape, size and color, (ii) complex external environments that can add difficulties when detecting a vehicle and (iii) their variable performance that can vary even depending on environmental conditions such as snow, fog and rain [27,28]. In this context, Fu et al. [29] propose integrating existing tracking and classification computer vision methods for achieving a data collection under low visibility conditions, at night time and in shadowed areas.

This paper adopts an alternative approach by generating O-D data based on induction loop detection, which requires reverse engineering efforts to estimate the points of origin and destination of a route. Additionally, and differently from previous works, we go one step beyond by showing how O-D matrices can be modified in order to emulate the behavior under different degrees of congestion.

## 3. Overview of the Simulation Tools Adopted

In this section, we detail our target traffic simulator, called SUMO [3]. Furthermore, since our focus is on generating an O-D matrix for SUMO based on induction loop detection data, we will also introduce the DFROUTER [6] tool.

### 3.1. SUMO Overview

Usually, traffic modeling consists of obtaining a few variables, such as time of departure, the route followed by the different vehicles and in some cases the duration of the route. Notice that the latter cannot be calculated in a very realistic way, since it assumes a certain vehicle, driver and state of traffic congestion along the route. SUMO [3] addresses this challenge through detailed microscopic modeling of cities and vehicles, offering open source packages for traffic simulation that have evolved into a full range of utilities. In fact, as an open source simulator, it is constantly being improved, and it is highly accepted among the scientific community. Its features include: simulation of multimodal traffic, traffic schedules, support to various map formats like OpenStreetMaps, ability to import road network maps in multiple formats and generating routes from multiple sources. It also offers high-performance simulation support through the TraCIinterface, allowing one to perform interactive simulations when combined with another simulator (e.g., OMNeT++ [30]).

A key advantage of SUMO is its multimodal simulation support, as it not only includes the movement of vehicles in a city, but it also includes the public transportation system, rail networks and even pedestrian paths. This means that a traffic modality can be described by multiple routes, which may be composed of several sub-paths.

Since the traffic flow is simulated microscopically, every moving vehicle within the network is modeled individually, being located at a specific position and associated with a specific speed. Each time step has a duration of one simulated second by default, which allows for the discrete simulation of continuous mobility in space. In addition, simulations consider the different attributes of roads, such as maximum speed or priority rules toward the right side, while also accounting for realistic driver models.

### 3.2. DFROUTER Operation

**Figure 1:**
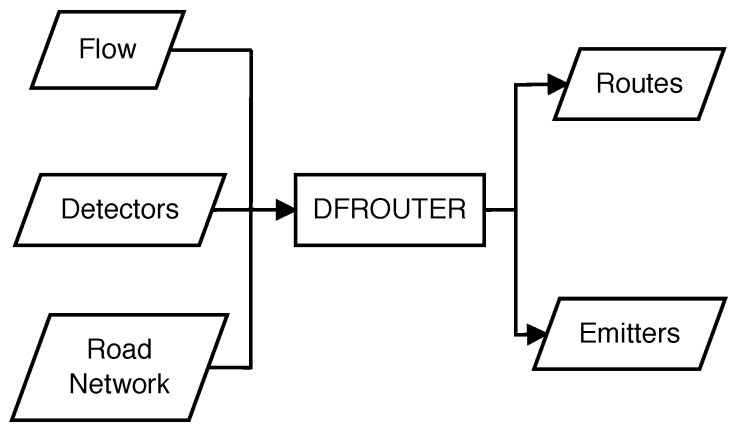
Flowchart of DFROUTER.

Among the packages included in the SUMO distribution Version 0.20.0, we rely on the DFROUTER [6] tool, which has been designed for road scenarios based on the idea that the roads are equipped with induction loops, measuring the inflow and outflow of the roads. DFROUTER is able to reconstruct the number of vehicles and routes in the simulation of the road network, from the information of types of vehicles, flows and speeds to achieve the desired O-D matrix. In particular, this tool allows, starting from induction loop counts for all the different roads of a city, estimating the actual vehicle routes that match such input. The algorithm that employs DFROUTER works if the network is partially or completely covered with the induction loops and generates paths by accounting for all the O-D pairs [7]. Figure 1 illustrates the different elements associated with this process. DFROUTER needs input data from flow detectors, in addition to the road network. Based on them, it will provide output data about routes, as well as the origin and destination for the different vehicles. The steps taken by DFROUTER are the following:

1. Importing the road network, including the detector positions and associated measurements.
2. Applying the detector classification for the following categories: source detectors (starting points of routes), in-between detectors and sink detectors (ending points of routes).
3. Calculate the vehicle flow between consecutive detectors.
4. Compute the route usage probabilities. Usually, measurements are provided on a per-lane basis, and they need to be summarized for each cross-section.

The main requirement of DFROUTER is that the road network must contain at least one induction loop detector on each main road segment; secondary streets without induction loops are nevertheless taken into consideration for route calculation. Other sources of traffic flow information that differ from standard induction loops are not supported. This means that DFROUTER needs a network containing a list of induction loop detectors, including their position and associated vehicle counts.

An induction loop is considered a source detector when there is no other detector on the same street, or on any foregoing street, meaning that vehicles are assumed to start new paths on those points. In-between detectors are the most common in cities, being required when we aim at maximizing the match between reference sensor data and simulated measurements. These detectors can also act as starting points of vehicles. Finally, sink detectors are used when there is no other detector on preceding streets or in streets that follow; unless strategic parts of the city are clearly known to act as traffic sinks, this type of detector can be discarded.

The route data generated by DFROUTER are based simply on proportions of flow on the divided edges. According to the authors of this tool, this method works similarly to a static O-D matrix estimation, but it does not work as an O-D estimator and cannot be compared to O-D estimation methods such as maximum entropy, generalized least square, Bayesian inference, minimum information, etc. [7]. However, there are methods with which the DFROUTER algorithm can be compared, including: (i) the equally-split O-D matrix, which assigns all destinations in equal proportion, but with implausible results; (ii) the proportional O-D matrix, where the attraction of any destination is a function of the number of trips ending in that destination, as this method is identical to the one adopted in DFROUTER; (iii) the iterative method, which balances input and output flows through an iterative tuning algorithm, being identical to DFROUTER; (iv) the gravity model, which manages a concept where the probability of very long and very short trips on the freeways is low, while in DFROUTER, it depends heavily on the treatment of external stations; and (v) the turning percentage, a method that is also identical to DFROUTER.

After obtaining the information concerning the vehicles’ entry and exit points of the network, the third step determines the routes for each of the origins and destinations. For this purpose, a file is generated detailing all the considered routes, but lacking information about the number of vehicles associated with each route. Afterward, DFROUTER combines the calculated routes with flow information taken from real induction loop sensors to determine the number of vehicles associated with each route. Accomplishing this task requires a file containing the following information: (i) detector id, (ii) initial time, (iii) number of vehicles driving over the detector and (iv) average speed for these vehicles.

On the last step, DFROUTER generates the actual O-D matrix, allowing one to store information about the total number of vehicles, together with their routes. With regard to vehicles, their routes start at any source detector location along the associated route.

## 4. Traffic Flow Generation Methodology

In this section, we describe the procedure followed when attempting to obtain an accurate O-D matrix for the city of Valencia, Spain, where nowadays such essential data still remain unavailable. Notice that many of the cities around the world rely on this method and often lack any other sources of data beyond it. The most critical data for simulation experiments to be representative is the number of vehicles that are injected into the network and their destination (O-D matrix). In this regard, the DFROUTER tool is able to generate data encompassing routes and the number of vehicles associated with each route. Although not strictly an O-D matrix, such a matrix can be generated based on these data.

Below we describe the methodology followed to accomplish our goal of obtaining realistic traffic flow data, starting with an analysis of DFROUTER’s output, followed by the proposed heuristic, gradually reducing the number of vehicles passing through the induction loops and re-adjusting the number of vehicles to be injected in each segment until approaching the reference traffic, which is then validated against real data.

### 4.1. DFROUTER Output Analysis

To evaluate how DFROUTER behaves using data from real induction loops, we use a database provided by Valencia’s Traffic Department using the induction loop detector counts for the different streets and avenues of the city as input data. For our study, we chose the month of November because it has no holiday periods and it has values relatively similar to other months. Based on the data obtained by Calafate et al. [5], we focused on a typical Monday, which is representative of traffic behavior at rush hour from 8:00 a.m.–9:00 a.m. on business days with 520 induction loop detectors deployed throughout the city.

To obtain the traffic characterization for our network in terms of vehicles injected and their respective routes, we followed the four-step procedure defined in Section 3.2 for the DFROUTER tool. In particular, the reference traffic flow data based on real induction loop measurements is used as a reference, being compared against the data regarding virtual induction loops following simulation measurements. After completing this process, we noticed some mismatches between the generated traffic and the original data, meaning that DFROUTER does not accurately match real traffic in the city of Valencia for the time period specified. In particular, the number of vehicles circulating in the city using DFROUTER is 138.9% greater than the overall number of vehicles detected by induction loops in the reference data. This happens because DFROUTER only knows the number of vehicles that pass through an induction loop detector, but it does not know the precise number of vehicles that have to be injected into the vehicular network to obtain that number. Furthermore, the traffic of different routes can converge to the same induction loop, and so, it becomes difficult to predict in advance the actual number of vehicles passing through the different induction loops without actually running the simulation. In the next subsection, we propose a heuristic to correct this error and provide a more realistic traffic characterization.

### 4.2. Proposed Iterative Heuristic

Considering the mismatch between DFROUTER’s output and real data described in the previous section, we now propose an adjustment heuristic to compensate this error and make the number of vehicles in our simulation environment as realistic as possible.

To achieve our goal, we propose a heuristic called “iterative” that regulates global traffic by affecting all streets in a proportional manner, thereby allowing one to adjust the traffic volume according to the reference data. In particular, since there are dependencies between induction counts on different streets and the traffic injected, the iterative process is required to find the best fit. The  proposed heuristic is shown in Algorithm 1.

As input data, we have the information concerning vehicular traffic at a specific time, available as detector counts, and the associated street segment ID.

An initial run of DFROUTER is required for obtaining routes and the O-D matrix using the reference induction loop data as input. The outcome of this first run returns an excess of 138.9% in terms of traffic volume, a value that is then refined using our proposed heuristic. In particular, it will adjust iteratively the number of vehicles injected in every street as provided to DFROUTER so that, when the output converges, the total number of vehicles accounted in the output of DFROUTER approaches the reference value. For this purpose, given a maximum number of iterations $n_{max}\in N$ and a maximum admissible error ε>0, we will build a succession of intervals ${\left\{\left[\varphi_{min}^{n},\varphi_{max}^{n}\right]\right\}}_{n=0}^{k}$ of traffic flow adjustment factors, with $k\leq n_{max}$ and $\left[\varphi_{min}^{n+1},\varphi_{max}^{n+1}\right]\subset \left[\varphi_{min}^{n},\varphi_{max}^{n}\right]\forall n$, as well as a succession ${\left\{\varphi_{n}\right\}}_{n=0}^{k}$ with $\varphi_{n}\in \left[\varphi_{min}^{n},\varphi_{max}^{n}\right]\forall n$, so that, for each iteration *n*, we will modify the number of vehicles injected on every street *s* as input to DFROUTER, and denoted by $\tau_{s,n}$, by relying on Equation (1):

(1) $$\tau_{s,n}=\left\lfloor \frac{\sigma_{s}}{\omega_{s}}\cdot \varphi_{n}\right\rfloor$$

where $\sigma_{s}$ is the smallest number of vehicles detected among all segments of the street *s*, $\omega_{s}$ is the number of segments that conform to street *s* and ⌊x⌋ represents the largest integer number less than *x*. This formula ensures that the load corresponding to a street, according to our induction loop data, is evenly distributed among the different segments on that street. Then, we apply the traffic flow adjustment factor $\varphi_{n}$ to each street segment of that street.

**Algorithm 1** Iterative heuristic.**Require:** Road Network, flow, detectors files, $n_{max}$ and ε**Ensure:** Vehicle-Street-Segment-info file1:α← calculate reference number of vehicles2:$\varphi_{min}^{0}\leftarrow 0$,$\varphi_{0}\leftarrow \varphi_{max}^{0}\leftarrow 1$,$\tau_{s,0}\leftarrow \left\lfloor \frac{\sigma_{s}}{\omega_{s}}\cdot \varphi_{0}\right\rfloor$3:Process input files with DFROUTER4:n←15:$\beta_{1}\leftarrow$ Vehicle count per street ID6:$\varphi_{1}\leftarrow \frac{\alpha }{\beta_{1}}$7:$\tau_{s,1}\leftarrow \left\lfloor \frac{\sigma_{s}}{\omega_{s}}\cdot \varphi_{1}\right\rfloor$8:Create a file with information about vehicles, segments and streets9:Apply $\tau_{s,1}$ to all street IDs ($\tau_{s,n}$)10:$\varphi_{min}^{1}\leftarrow \varphi_{min}^{0}$, $\varphi_{max}^{1}\leftarrow \varphi_{max}^{0}$11:**while** $|\frac{\beta_{n}}{\alpha }-1|>\varepsilon$ **and** $n<n_{max}$ **do**12:  Process input files with DFROUTER13:  n←n+114:  $\beta_{n}\leftarrow$ Vehicle count per street ID15:  **if** $|\frac{\beta_{n}}{\alpha }-1|>\varepsilon$ **then**16:    **if** $\beta_{n}>\alpha$ **then**17:      $\varphi_{max}^{n}\leftarrow \varphi_{n-1}$,$\varphi_{min}^{n}\leftarrow \varphi_{min}^{n-1}$18:    **else if** $\beta_{n}<\alpha$ **then**19:      $\varphi_{max}^{n}\leftarrow \varphi_{max}^{n-1}$,$\varphi_{min}^{n}\leftarrow \varphi_{n-1}$20:    **end if**21:    $\varphi_{n}\leftarrow \frac{\varphi_{max}^{n}+\varphi_{min}^{n}}{2}$22:    $\tau_{s,n}\leftarrow \left\lfloor \frac{\sigma_{s}}{\omega_{s}}\cdot \varphi_{n}\right\rfloor$ to all street IDs ($\tau_{s,n}$)23:  **end if**24:**end while**


As the initial step in our iterative algorithm, we assign initial values to the different variables involved as detailed in Equation (2):

(2) $$\begin{array}{cc} \varphi_{0}=1,\;\tau_{s,0}=\frac{\sigma_{s}}{\omega_{s}}\cdot \varphi_{0},\;\varphi_{min}^{0}=0, & \;\varphi_{0}=\varphi_{max}^{0}=1 \end{array}$$

Then, a relationship between the number of vehicles per street ID and the reference value (φ) is calculated according to Equation (3):

(3) $$\varphi_{1}=\frac{\alpha }{\beta_{1}}$$

where α is the real number of total vehicles passing through all street IDs (reference value) and $\beta_{1}$ is the simulated number of total vehicles passing through all street IDs, as provided by the initial execution of DFROUTER. In our case, α= 409,499, $\beta_{1}=$ 978,339, and therefore, $\varphi_{1}=0.41857$ (see Table 1). Note that we will always have $\alpha \leq \beta_{1}$, although the value of β should converge to α.

For the iterations that follow (n>1), the following equation applies:

(4) $$\varphi_{n-1},\;\tau_{s,n-1}=\frac{\sigma_{s}}{\omega_{s}}\cdot \varphi_{n-1}$$

We then proceed to execute DFROUTER in a loop by successfully refining the input values and obtain as output the total number of vehicles per street ID ($\beta_{n})$. The process continues converging until the error is close to zero by obtaining a sequence of values for $\varphi_{max}^{n}$ and $\varphi_{min}^{n}$ based on the relationship between α and β, so that, if $\beta_{n}>\alpha$, we consider that $\varphi_{max}^{n}=\varphi_{n-1},\varphi_{min}^{n}=\varphi_{min}^{n-1}$, and in the opposite case, if $\beta_{n}<\alpha$, we instead consider that $\varphi_{max}^{n}=\varphi_{max}^{n-1},\varphi_{min}^{n}=\varphi_{n-1}$. Then, a new $\varphi_{n}$ value is obtained for the next iteration using Equation (5):

(5) $$\varphi_{n}=\frac{\varphi_{min}^{n}+\varphi_{max}^{n}}{2}$$

A new value of $\tau_{s,n}$ (see Equation (1)) can then be derived and used as input to DFROUTER. The new β value following each iteration *n*, denoted as $\beta_{n}$, will be considered final when the error ($\frac{\beta_{n}}{\alpha }-1$) is below ε, or the maximum number of iterations is reached, as stated in Equation (6):

(6) $$|\frac{\beta_{n}}{\alpha }-1|<\varepsilon \;or\;n=n_{max}$$

**Figure 2:**
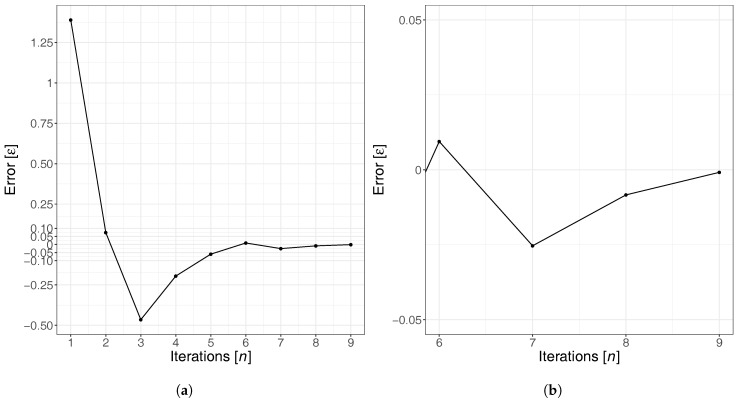
Estimation error evolution when increasing the number of iterations. (**a**) Full chart; (**b**) zoom for near zero error values.

Experimental results in Table 1 show that, initially, the value of $\frac{\beta_{n}}{\alpha }-1$ will fluctuate between positive and negative values, until convergence near zero is achieved, as illustrated in Figure 2. For instance, from Table 1, we have that the DFROUTER output value $\beta_{2}$ is 439,738, which is greater than α, and $\frac{\beta_{n}}{\alpha }-1=0.07384$. Since we want a small error, from interval $\left[\varphi_{min}^{1},\varphi_{max}^{1}\right]\;=\left[0,1\right]$ and $\varphi_{1}$ = 0.41857, the heuristic creates interval $\left[\varphi_{min}^{2},\varphi_{max}^{2}\right]=\left[0,0.41857\right]$ and value $\varphi_{2}$ = 0.20928.

Convergence is guaranteed in the scope of the proposed iterative heuristic, as we prove next:

On the one hand, we have that:

$$\forall_{n}\in N\;\;\left[\varphi_{min}^{n+1},\varphi_{max}^{n+1}\right]\subset \left[\varphi_{min}^{n},\varphi_{max}^{n}\right]$$

Moreover,

$$\forall_{n}\geq 2\;\;\;\;0<|\varphi_{max}^{n+1}-\varphi_{min}^{n+1}|=\frac{1}{2}|\varphi_{max}^{n}-\varphi_{min}^{n}|$$

$$=\frac{1}{2^{n-1}}|\varphi_{max}^{2}-\varphi_{min}^{2}|\leq \frac{1}{2^{n-1}}$$

Therefore,

$$\begin{array}{c} 0\leq \lim_{n\to \infty }|\varphi_{max}^{n+1}-\varphi_{min}^{n+1}|\leq \lim_{n\to \infty }\frac{1}{2^{n-1}}=0 \end{array}$$

which implies that:

$$\begin{array}{c} \exists \lim_{n\to \infty }\varphi_{min}^{n}=\lim_{n\to \infty }\varphi_{max}^{n} \end{array}$$

On the other hand, $\varphi_{n}\in \left[\varphi_{min}^{n},\varphi_{max}^{n}\right]\;\forall_{n}\in N$. Consequently, applying the sandwich criterion, we have that:

$$\begin{array}{c} \exists \lim_{n\to \infty }\varphi_{n}=\lim_{n\to \infty }\varphi_{min}^{n}=\lim_{n\to \infty }\varphi_{max}^{n} \end{array}$$

which in turn implies that:

$$\begin{array}{c} \forall_{s}\;\;\;\exists \lim_{n\to \infty }\tau_{s,n}=\left\lfloor \lim_{n\to \infty }\frac{\sigma_{s}}{\omega_{s}}\cdot \varphi_{n}\right\rfloor =\left\lfloor \frac{\sigma_{s}}{\omega_{s}}\cdot \lim_{n\to \infty }\varphi_{n}\right\rfloor \end{array}$$

Note that values $\tau_{s,n}$ must be integers because they represent numbers of vehicles. Therefore, the existence of the above limits implies that $\exists n_{0}\in N$ such that $\forall_{n}\geq n_{0}$ and $\forall s\;\;\tau_{s,n}=\tau_{s,n_{0}}$. That is, from iteration $n_{0}$, the input data to DFROUTER are always the same, and as DFROUTER is deterministic, its output data will also be always the same. This guarantees the existence of $\lim_{n\to \infty }\beta_{n}$, which does not necessarily coincide with α because both α and the limit are integer values, but according to the construction of the successions in the algorithm, it must be very close to α. Hence, given a reasonable upper bound to the number of iterations $n_{max}$ and a reasonable error ε>0, it is expected that for some $n\leq n_{max}$, $\begin{array}{c} \frac{\beta_{n}}{\alpha }-1 \end{array}<\varepsilon \;$. Otherwise, the algorithm will stop in value $\beta_{n_{max}}$.

When the execution of Algorithm 1 ends, we generate two files that contain traffic information associated with the various street segment IDs. The first one is composed by a tuple of two elements, where each tuple includes: (1) street segment ID and (2) the number of vehicles per segment ID. The second file aggregates different street segment IDs belonging to the same street, and it includes a set of three elements: (1) street ID, (2) associated segment ID and (3) segment ID with the lowest number of vehicles, where the latter corresponds to the number of vehicles injected that start on that particular street.

### 4.3. Goodness of Fit Achieved by the Proposed Heuristic

**Figure 3:**
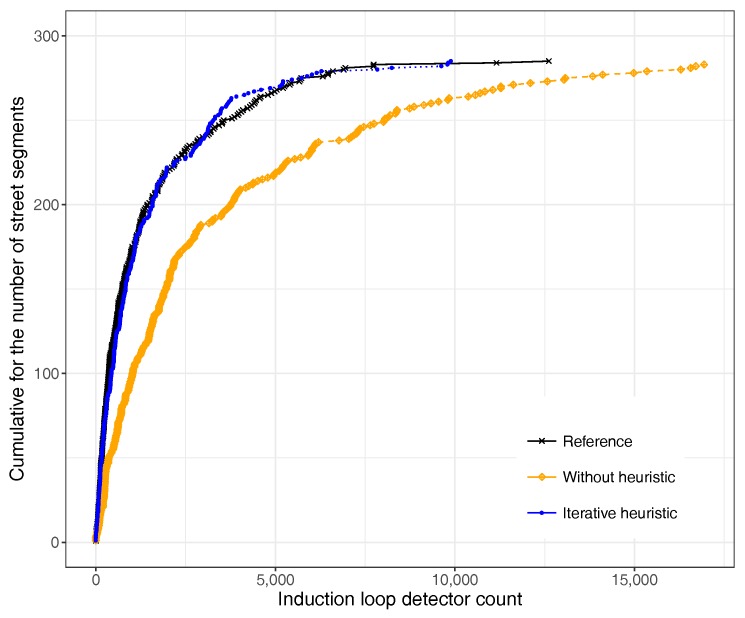
Adjustment of vehicles in Valencia using the proposed heuristic.

The methodology described above basically consisted of using our reference induction loop data as input to DFROUTER and then refining output values in an iterative manner until the overall number of vehicles for the reference and that for the generated data are quite similar. To validate the adequacy of this methodology and check if the global optimization approach was actually traduced in a similar distribution of values at finer levels of granularity, we obtained the cumulative distribution function of traffic for the different streets segments in the target city area. Figure 3 shows the obtained results, where we can observe that indeed the outcome of the iterative algorithm has a high resemblance with the reference data, achieving a behavior similar to the desired one in terms of the number of vehicles passing through the detectors for the different street segments of the city.

## 5. Simulation Results

In this section, we detail the tests and simulations made for the three tested cities: Valencia, Cologne and Bologna. For each city, we evaluate the traffic source distribution, the traffic destination distribution and the traffic dispersion distribution. Notice that, for the city of Valencia, we are using traffic flow definitions obtained according to the iterative heuristic defined in Section 4.2 with φ=0.38914 and ε<0.0001 in the ninth iteration, while the latter two are typical scenarios provided by the SUMO tool itself that will be used as a reference. In particular, we want to determine whether the effectiveness of our proposed approach allows generating O-D and route information comparable to Cologne and Bologna, or if, on the contrary, results differ too much from these two reference cities.

Table 2 shows the main characteristics of the three different cities analyzed. In terms of target area, we find significant differences. In the case of Bologna, the target area is only 2.34 km${}^{2}$. For Cologne, we have the opposite situation, where the target area has a size of 595.9 km${}^{2}$. In the case of Valencia, the area analyzed covers the whole city (excluding suburban areas), having a size of 77.43 km${}^{2}$ (intermediate case compared to others). In terms of street segment densities, they are clearly correlated with the overall area. If focusing on the average number of segments per km${}^{2}$, though, we find that the density for Valencia and Bologna is quite similar, while Cologne has a much lower density since suburban areas are also included in the map, as well. Concerning vehicle density, this is a metric that again has a clear relationship with the target area. In the case of Bologna, only a traffic-intensive area is analyzed. Cologne is in the opposite situation, covering a vast area, and for Valencia only, the whole city is included, while suburban areas are omitted.

Focusing on the generated traffic for the city of Valencia, the required information was obtained according to the procedure defined in Section 4. In the case of Cologne, data were generated by the German Aerospace Center (DLR) [31] using the SUMO mobility simulator. In particular, they relied on the DUAROUTER [32] tool to obtain routes through shortest path computation. Regarding the city of Bologna, its data were obtained from induction loops, as well, as supplied by the Municipality of Bologna. For performing route calculations, they get new routes assigned randomly according to a given distribution [33].

Our analysis focuses on three different metrics: traffic sources’ distribution, traffic destinations’ distribution and traffic dispersion distribution. In addition, for each of these metrics, we will analyze:

- The vehicle density per km${}^{2}$.
- Percentage of street segments affected.
- The distribution along a map.
- The Cumulative Distribution Function (CDF) of vehicles per segment.

For the sake of a fair comparison, in the experiments that follow, all scenarios were simulated using a simulation time of 900 s in their respective peak hours.

We start our analysis by studying the location of the different traffic sources, their density per km${}^{2}$ and how vehicles’ starting points (i.e., sources) are distributed throughout the city map.

**Figure 4:**
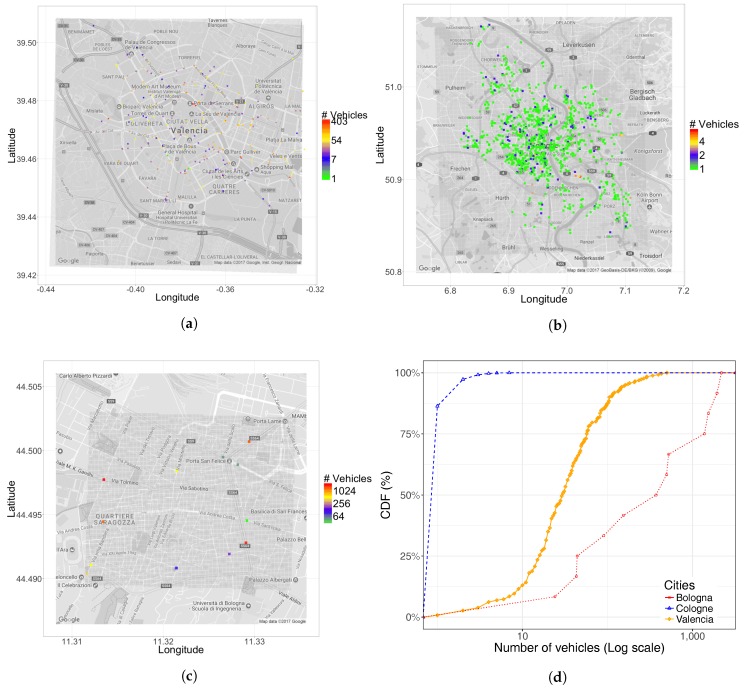
Geographical distribution of traffic sources (**a**–**c**) and CDF for number of vehicle per traffic source. (**a**) Valencia; (**b**) Cologne; (**c**) Bologna; (**d**) CDF.

As shown in Table 3, Bologna has a vehicular density that is higher than other cities; however, notice that only a very small and busy area of the city is studied, as shown in Figure 4c. For Cologne, we have the opposite situation compared to Bologna; Table 3 shows that it has a very low density of departure points, although we can observe a higher number of points in Figure 4b; this difference is expected as the target area is much greater, including more peripheral zones. Concerning Valencia, the number of traffic sources per square kilometer is a value in-between both reference cities. Figure 4a shows that these values are distributed throughout the city in a relatively homogeneous manner. Overall, we find that, in all cases, the number of source positions tends to be quite small.

Regarding the number of vehicles associated with each particular departure point, Figure 4d shows that, in Cologne, the number of vehicles associated with each source is in general very small (always less than 10); on the contrary, in Bologna, each traffic source may inject up to several thousand vehicles, which seems perhaps unexpectedly high. For Valencia, the spectrum of possible situations is much wider, where most traffic sources inject a moderate amount of vehicles (between 10 and 500 vehicles), including source points with only two or three vehicles. Overall, the obtained results for Valencia are more representative of a real metropolitan area, where highways may inject a high vehicle load into the system, while vehicles may also depart from other parts of the city, as well.

### 5.1. Traffic Destination Analysis

We now study the location of the different traffic destinations, their density per km${}^{2}$ and how vehicles associated with the different ending points vary.

**Figure 5:**
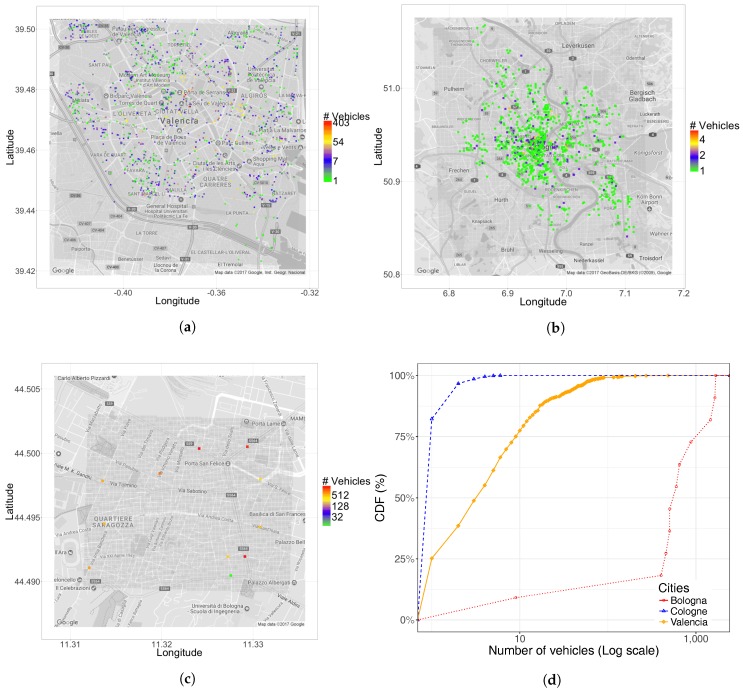
Geographical distribution of traffic destinations (**a**–**c**) and CDF for the number of vehicles per destination position. (**a**) Valencia; (**b**) Cologne; (**c**) Bologna; (**d**) CDF.

As shown in Table 4 and Figure 5, we find that Valencia is clearly the one offering a richer set of destinations. In addition, although traffic destinations for Cologne and Bologna slightly decrease with respect to the number of sources, for Valencia, this value substantially increases. This means that, for the specific case of Valencia, vehicle sources are often concentrated at some specific positions, like highway entrances, but then, the destinations of those vehicles can vary substantially, as occurs in real life, which is indeed a good result.

Regarding the CDF for vehicle destinations, Figure 5d shows that Cologne maintains a distribution similar to the one observed for traffic sources (see Figure 4), while for Bologna, a trend to concentrate destinations is detected (right shift).

Focusing on Valencia, we find that there is a clear left shift, which means that vehicles tend to complete their routes in a more heterogeneous manner, in accordance to the indexes and observations referred to above, while maintaining locations that concentrate vehicle destinations. As expected, these locations are associated with the main highway exits.

### 5.2. Traffic Dispersion Analysis

Our first analysis concludes by studying traffic dispersion. In particular, we determine how the different street segments that conform to the target maps are used by vehicles. For experiments to be meaningful, most of the main streets/avenues should have a non-zero traffic flow. Therefore, we study the density of occupied street segments per km${}^{2}$ throughout the simulation time.

**Figure 6:**
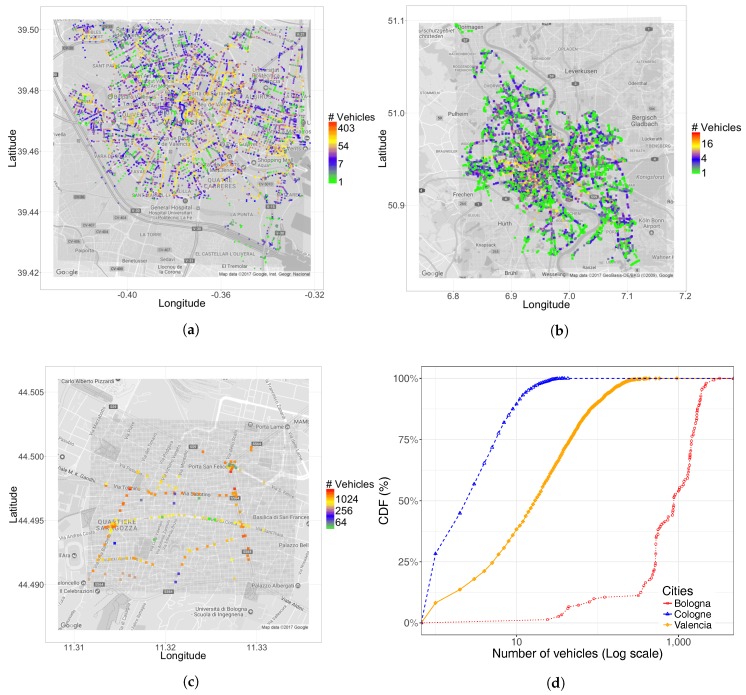
Geographical distribution of the street segments occupied by traffic (each point represents one full segment) and the CDF for the number of vehicles per street segment throughout the experiment (**d**). (**a**) Valencia; (**b**) Cologne; (**c**) Bologna; (**d**) CDF.

Focusing on the number of occupied street segments per km${}^{2}$, both Table 5 and Figure 6 show that Valencia achieves a better occupation, which is associated with a better traffic distribution. For Cologne, we have the opposite situation the number of occupied street segments per squared kilometer being rather low. In addition, we find that the majority of street segments remain unused (65.03%). For Valencia, this value is much lower (35.52%), in accordance with the percentage of secondary streets having negligible traffic. Regarding Bologna, its values are in a mid-range compared to Valencia and Cologne. Concerning the CDF for the number of vehicles per street segment throughout the experiment, Figure 6d shows that, similarly to the CDF for traffic sources (see Figure 4d), Valencia is characterized by a wider range of situations, contrary to Cologne (traffic is very sparse) and Bologna (traffic is very dense). In addition, if we compare the difference in shape between Figure 4d and Figure 6d, we observe that there is a general trend of widening the range of values and shifting towards the central values. Therefore, in the case of Cologne and Bologna, we can now detect some traffic concentration effects (right shift of the curve), while in Valencia, we have simultaneous traffic concentration and traffic dispersion effects, since the range of values now increases.

Overall, the analysis made shows that the procedure followed to generate O-D matrices for the city of Valencia, along with the mobility simulation to retrieve routes and the corresponding street occupation, clearly evidence that the results produced are exactly in-between both scenarios used as a reference, as expected, being in accordance with the target area. In particular, they keep a clear relationship with the results for reference cities with wider areas (Cologne) and narrower areas (Bologna). Regarding the flows that are generated, we find that for Valencia, we achieve a much richer result, being that more possible flow destinations per squared km are contemplated and that the percentage of segments used as destinations are also significantly greater that for the two other cities.

## 6. Traffic Congestion Analysis

In the previous sections, the traffic load for the city of Valencia had been properly adjusted and contrasted with other existing cities, allowing one to achieve an O-D matrix that represents real traffic conditions at a specific day/time [4]. However, traffic planning and optimization typically require studying the impact of varying these conditions, especially if attempting to determine what degree of congestion is expectable if certain conditions cause additional traffic to circulate in the city. With this aim in mind, we will first detail how, based on that reference scenario, we can regulate the amount of vehicles in the city $\left(\lambda \right)$ to generate different degrees of congestion having the flexibility to congest the city according to any criteria. Specifically, our approach consists of attempting to inject an extra number of vehicles on each street segment (see Equation (7)).

(7) $$\tau_{s,n}=\frac{\sigma_{s}}{\omega_{s}}\cdot \varphi_{n}+\lambda$$

Once the simulation is executed under the new parameters, we then check the actual number of additional vehicles available in the system during the experiment, because not all vehicles can actually be injected if congestion is too high.

Since our control over additional traffic has a per-street granularity, below, we detail two different methods for varying the traffic load and study for each of them the impact of traffic congestion on different metrics of interest, including average vehicle speed, average travel time and the ratio of vehicle arrivals.

### 6.1. Uniform Traffic Load Regulation

In this section, we study the impact of uniformly varying the traffic load throughout the city of Valencia. To achieve this goal, our procedure was based on the reference traffic scenario for Valencia derived in Section 4, to assign a variable number of additional vehicles to be injected at each traffic source location.

Overall, we performed tests by varying the total number of additional vehicles injected into the system from 2271–34,065.

Concerning the average vehicle speed metric, it was obtained by performing an arithmetic average of the speeds of the vehicles throughout the simulation experiment.

**Figure 7:**
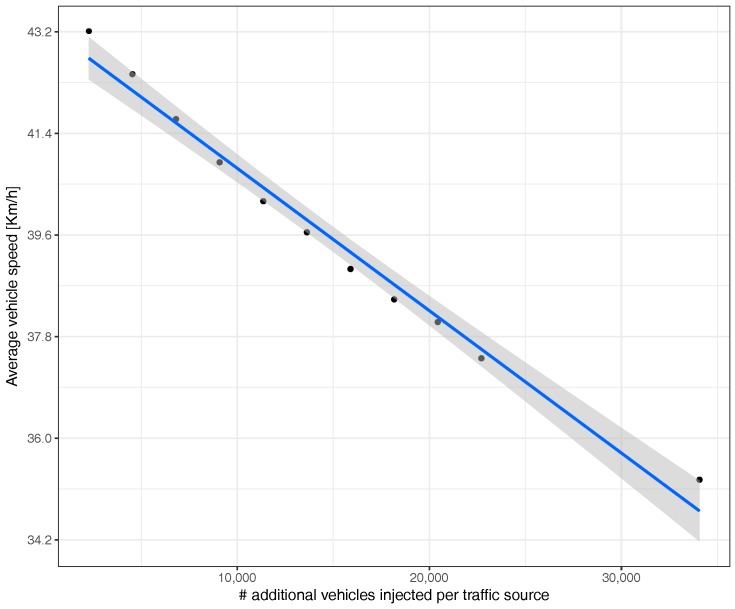
Average travel speed when uniformly varying the traffic volume.

Figure 7 shows that, as expected, increasing the number of vehicles injected causes the average speed of vehicles to decrease due to the traffic jams that start to build up on the different routes. Furthermore, notice that, within the range of values tested, the correlation obtained is linearly inverse, meaning that congestion adjustments can be done in a straightforward manner.

**Figure 8:**
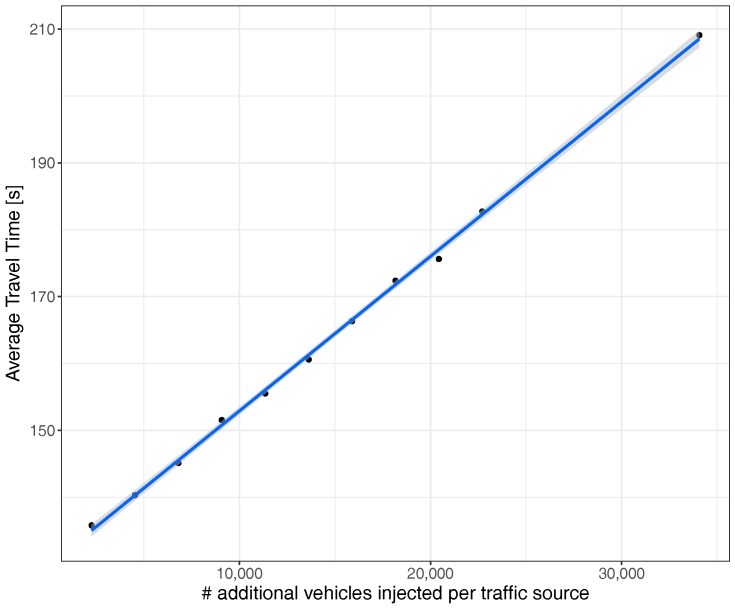
Average travel time when uniformly varying the traffic volume.

We now proceed to study the average travel time of vehicles that have reached their destination; vehicles that failed to reach their destination during the simulation time are excluded from our results. As shown in Figure 8, the average travel time increases in a linear fashion as traffic in our scenario also increases. This trend is expectable and very similar to real traffic conditions according to HCM [34].

**Figure 9:**
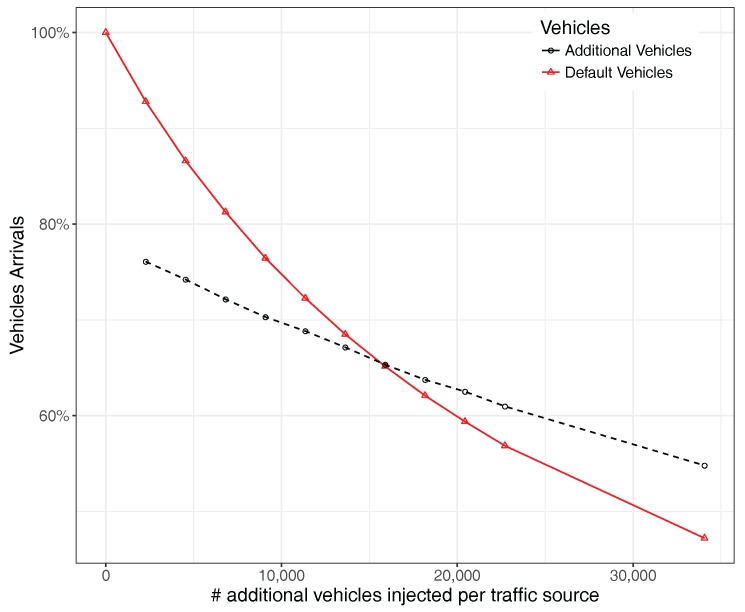
Vehicles arrivals (in percentage) when uniformly varying the traffic volume.

Regarding vehicle arrivals, Figure 9 shows that, when injecting more vehicles in the road network of the city, all groups of vehicles (both default and additional ones) experience a drop in their arrival ratio to their respective destinations, this decay being more pronounced for the default group of vehicles, as their number is actually larger.

**Figure 10:**
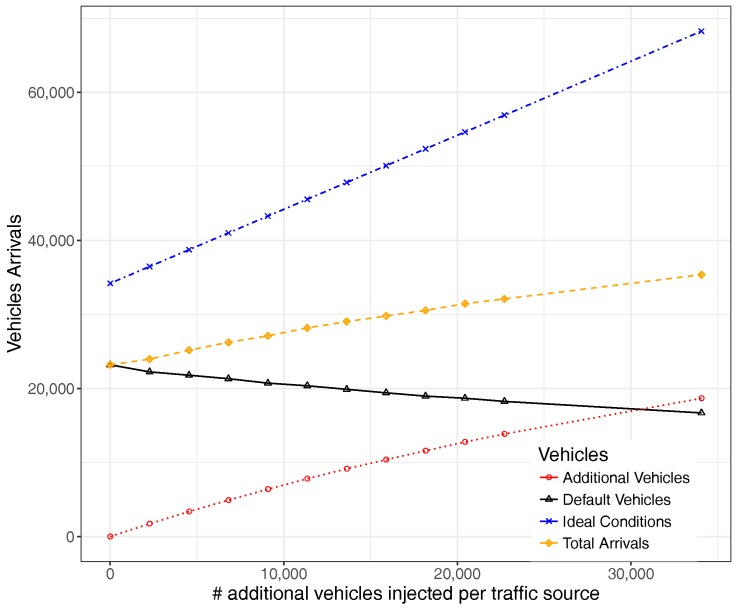
Vehicle arrivals.

Furthermore, when focusing instead on the absolute value of vehicles, Figure 10 shows that the number of vehicles in the default group experiences a constant drop, while additional injected vehicles experience an increase whose arrival rate slope slows down when reaching saturation conditions (number of additional vehicles injected similar to the default number of vehicles).

It is also worth observing how the total number of vehicles in the system varies, as well as its difference towards the ideal case, a situation where the network capacity and conditions would allow all vehicles to reach their destination in a minimal time. Thus, the number of vehicles not arriving at their destinations is given by the difference between these two lines (ideal vs. total), a number that tends to increase more and more as system saturation conditions are reached.

To complete our analysis of the results obtained, we now present the correlation between travel time and travel speed, followed by a travel time vs. travel distance analysis. Such correlation analysis aims at providing a greater insight into traffic behavior under congestion.

**Figure 11:**
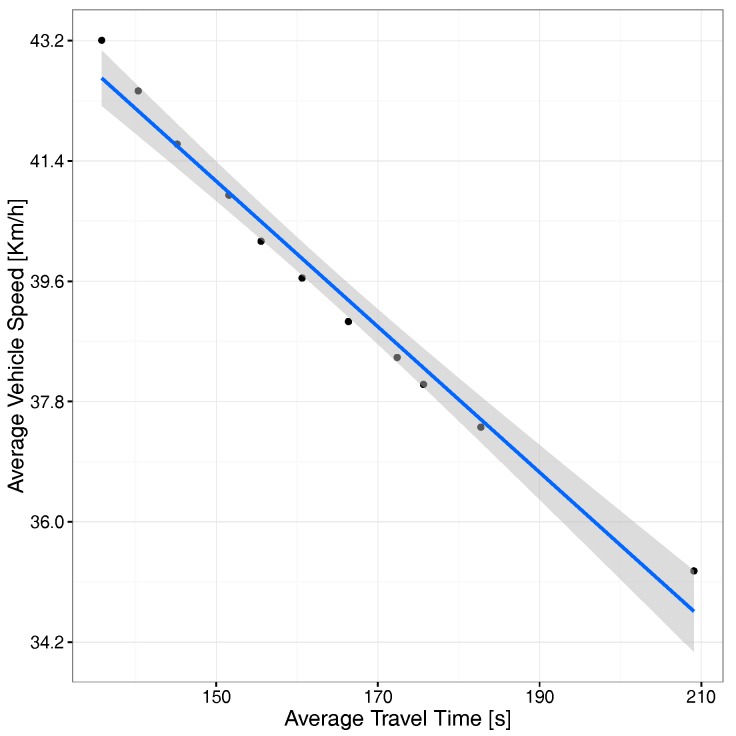
Correlation between average travel time and average speed.

In terms of travel time vs. travel speed, Figure 11 shows that, when the average travel time increases, the vehicles’ travel speed decreases, a situation expectable for scenarios gradually becoming congested due to the presence of an excessive number of vehicles on the different streets.

**Figure 12:**
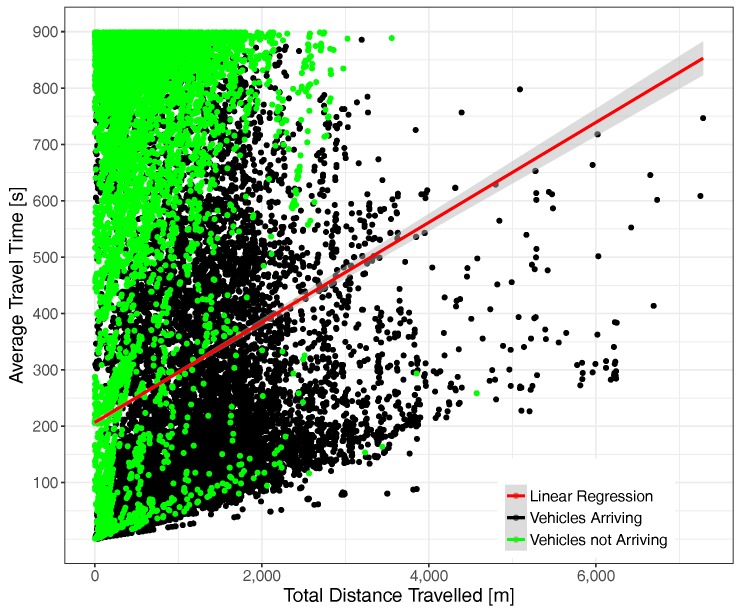
Time-distance correlation under moderate traffic load conditions (total number of vehicles = 30,871).

Focusing now on the correlation between the actual distance traveled by vehicles according to their total time in the scenario, Figure 12 illustrates the behavior experienced under moderate traffic load conditions. As expected, distance and total time are clearly correlated, although we observe that the actual situation is quite complex, being that vehicles not arriving to their destinations were, in most cases, injected into the scenario in the initial simulation period, typically traveling a relatively small distance during that time. Concerning vehicles arriving at their destination, we find they were in some cases able to travel for long distances (>7 km in some cases), although shorter trips are obviously prevalent. It is also worth pointing out that the maximum speed limits impose a minimum time to traverse a certain distance, which explains the slope of the points at the bottom of the chart.

**Figure 13:**
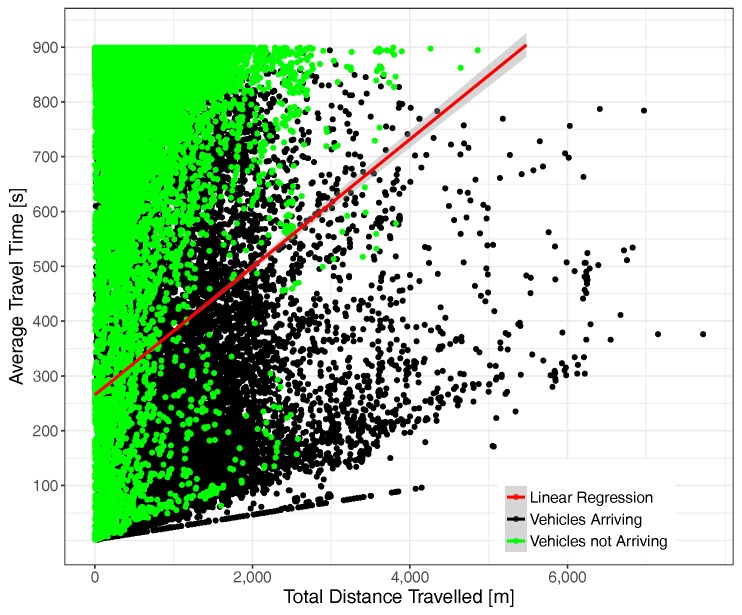
Time-distance correlation under traffic saturation conditions (total number of vehicles = 53,463).

Figure 13 depicts the situation under high traffic load conditions. We find that, on the one hand, point density has significantly increased (since each point represents a vehicle) and also that the correlation between time and distance now have a higher slope, meaning that more time is required, on average, to travel the same distance. Concerning the point distribution corresponding to vehicles arriving and not arriving at their final destination, we observe that both tend to concentrate nearer to the Y axis, a phenomenon that is expectable due to congestion, although again, we observe several cases of vehicles able to travel long distances.

### 6.2. Hotspot-Based Traffic Load Regulation

**Figure 14:**
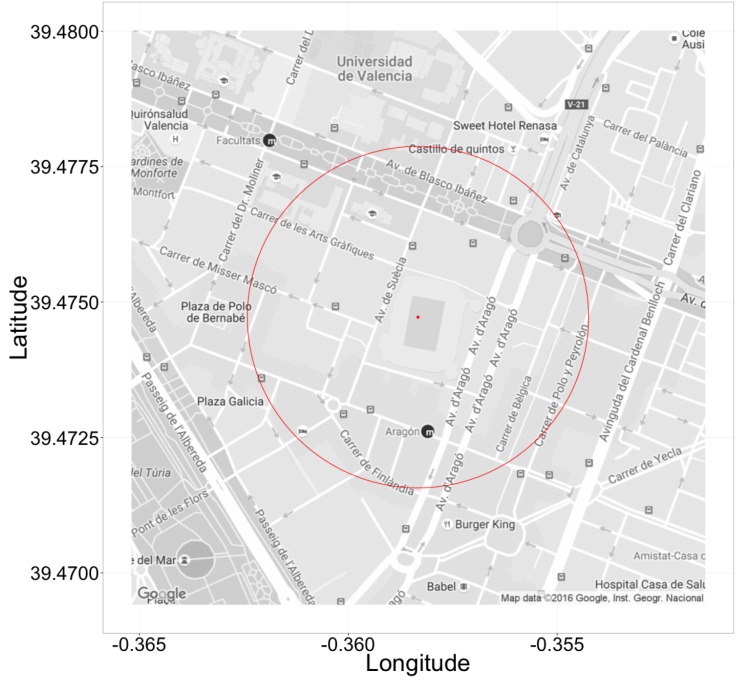
Area of our hotspot-based traffic.

In this section, our focus is now pointed towards situations where a public event is able to attract a high number of people to a very restricted area (e.g., football game), thus causing the city to experience a heterogeneous congestion effect. With this goal in mind, the Mestalla Stadium was chosen as the hotspot for our scenario. Notice that it is the biggest stadium in the city of Valencia with a capacity of 54,000 spectators. Additionally, we find that up to 106 different routes pass near this stadium (within a 270-m radius). Therefore, our strategy was to gradually inject vehicles into the system from nearly 100 to nearly 10,000; such vehicles depart from this area to congest traffic as though it were a real situation taking place right after a sports event (see Figure 14). Notice that the additional vehicles will depart from the stadium to various end routes, thereby mixing with other existing traffic throughout the entire city.

**Figure 15:**
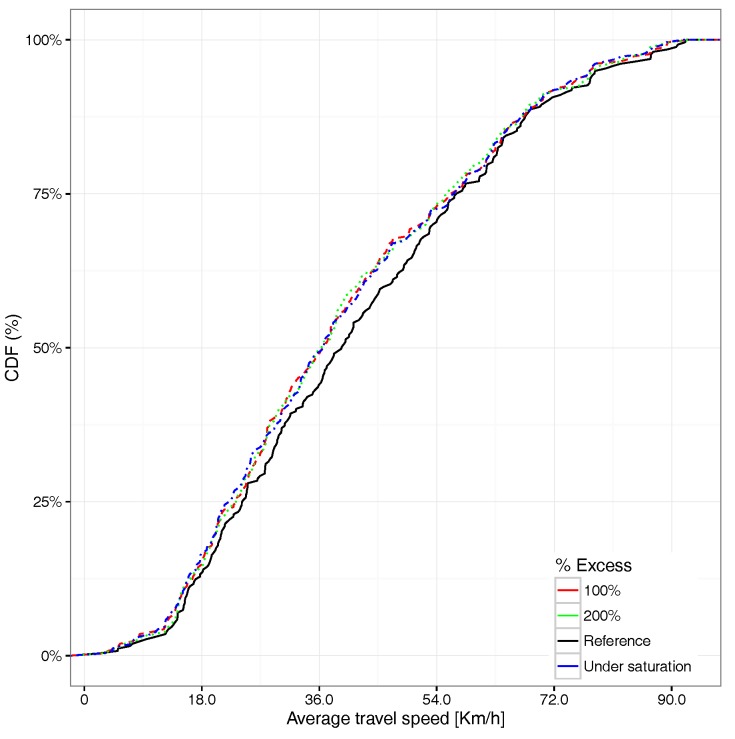
CDF for the average travel speed under different saturation levels.

Starting by observing the average travel speed distribution for all vehicles, Figure 15 shows that injecting more vehicles causes the average travel speed to be reduced; in particular, about 70% of the vehicles experience a travel speed below 54km/h.

**Figure 16:**
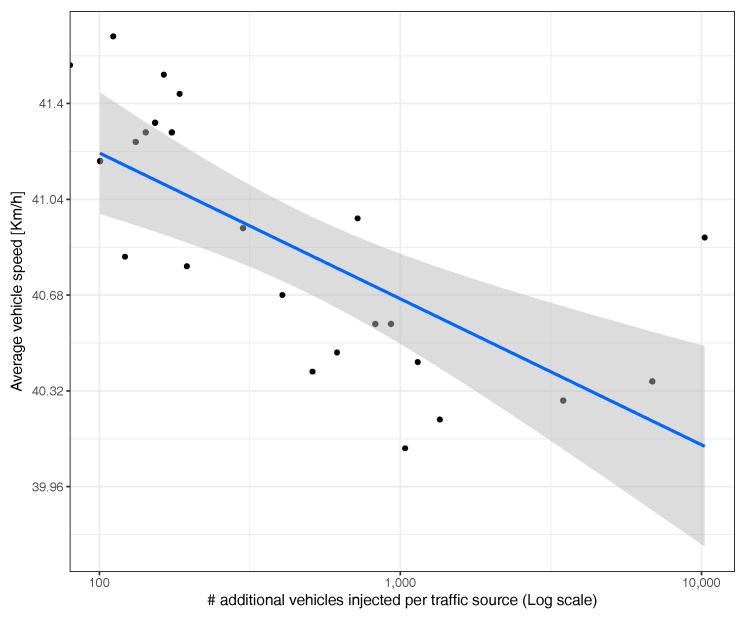
Average vehicle speed in different experiments.

Figure 16 shows instead the arithmetic average speed of all vehicles when increasing the number of additional vehicles injected into the system. We find that, as the number of vehicles inserted in the network increases, the average vehicle speed tends, in general, to decrease, although the degree of variability between experiments is high, as evidenced by the confidence envelope (represented in the figure as a gray shadow).

**Figure 17:**
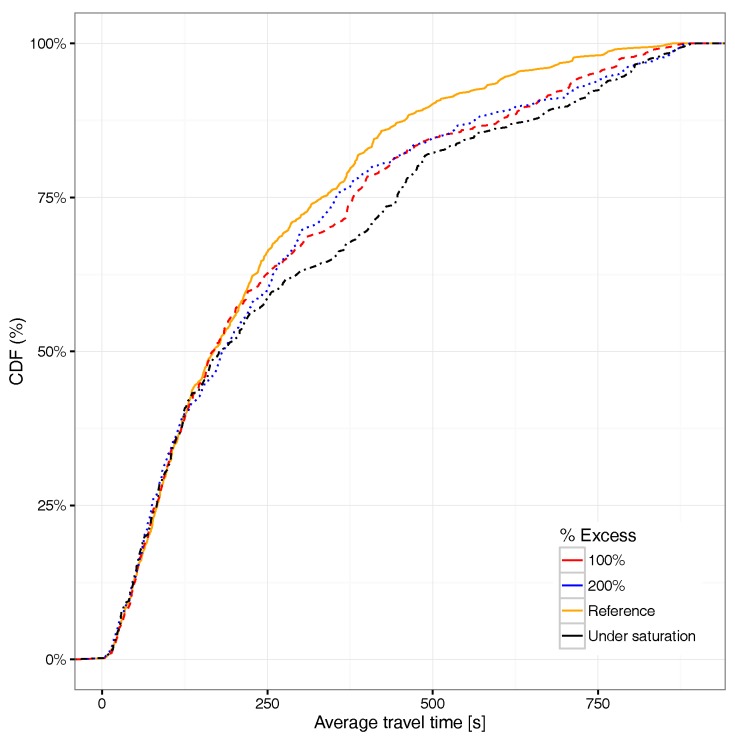
CDF for average travel time under different saturation levels for vehicles arriving at their destination.

**Figure 18:**
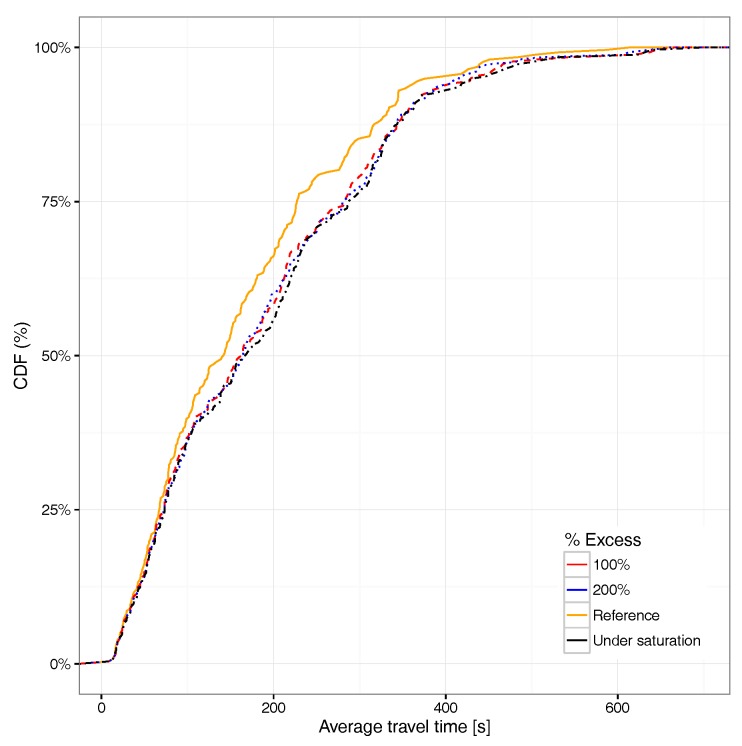
CDF for average travel time under different saturation levels for all vehicles.

Focusing now on the average travel time associated with the different vehicles able to reach their destination, Figure 17 shows that an excess of 100% in the number of vehicles already has a quite significant impact on the average travel time distribution, the curve being in some cases very close to the saturation situation. Compared to the travel time CDF for all the vehicles (see Figure 18), we find that the overall differences compared to the reference case (non-congested) are basically the same for all saturation cases, meaning that the impact with 100% more vehicles departing from the hotspot area is already significant. If we compare Figure 17 against Figure 18, we find that the differences for the former are more accentuated with respect to the latter. This occurs since, when considering all vehicles and not only those reaching their destination, we include many more vehicles, including those generated near to the end of the simulation, which in general, are unable to reach their destination, which explains why differences become more subtle.

**Figure 19:**
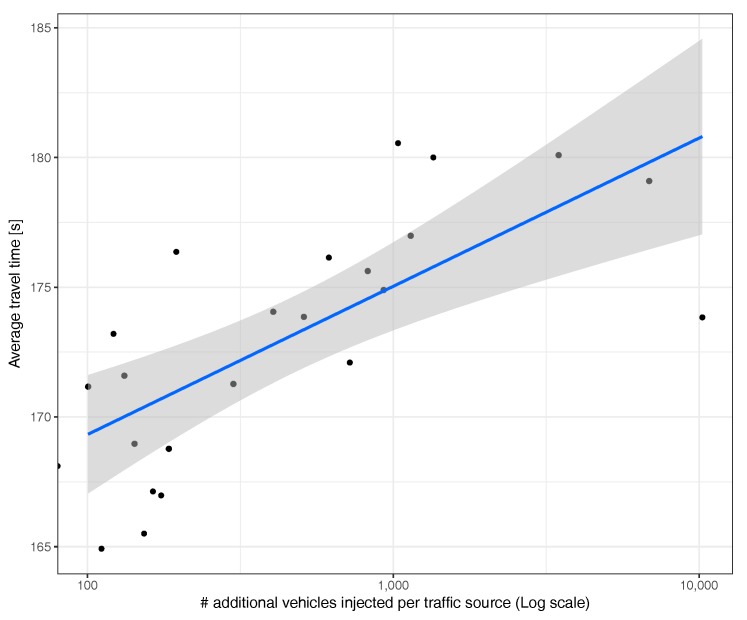
Average travel time in different experiments.

Figure 19 summarizes the correlation between additional injected vehicles and average travel time, evidencing that hotspot-based traffic injection is only able to provoke a moderate increase on the overall travel time of vehicles, as the impact in areas far away from the congestion point remains rather limited.

**Figure 20:**
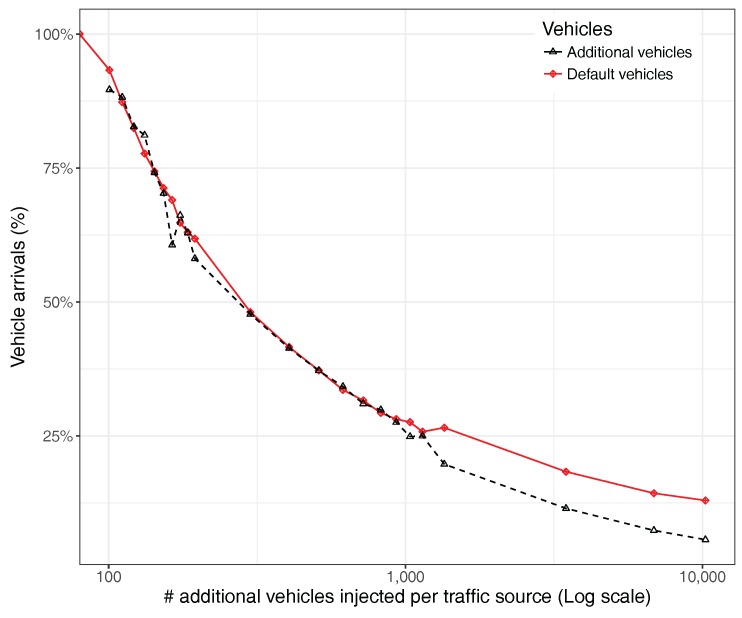
Vehicle arrival ratio when varying the number of additional vehicles injected per traffic source.

We now concentrate on the vehicle arrival ratio to see the percentage of vehicles that fail to complete their route within the simulation time. Figure 20 shows that, as the number of injected vehicles increases, the overall vehicle arrival ratio will experience a consistent decrease. When reaching more than 1000 additional vehicles injected, though, the penalty experienced by additional vehicles is higher compared to the default vehicles in the scenario. This means that the congestion near the hotspot tends to become critical, but the impact on the rest of the traffic is not so dramatic.

**Figure 21:**
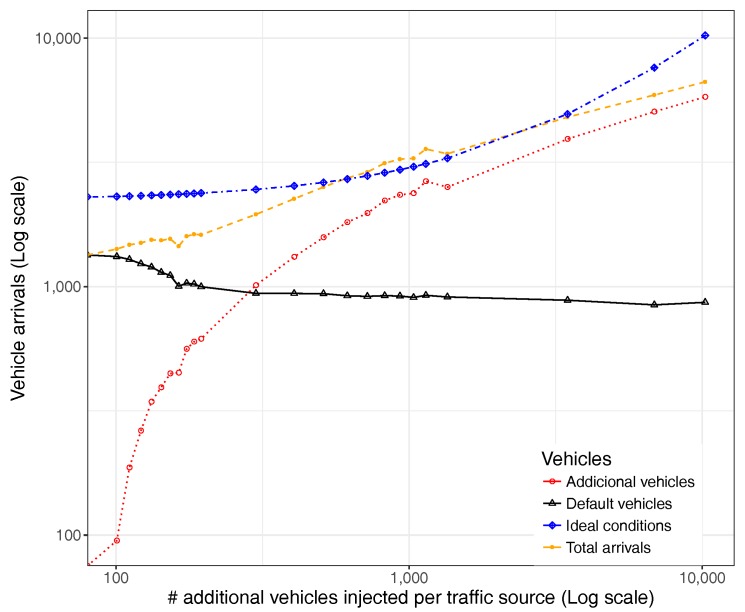
Vehicle arrival ratio when varying the number of additionally injected vehicles.

Figure 21 provides further insight by representing the actual number of vehicles that arrive at their destination along with the total number of arrivals and the maximum theoretical case under ideal conditions. We can see that, as we increase the traffic load, the overall number of vehicle arrivals is able to increase only up to a certain extent (about 9000 vehicles), beyond which saturation is reached. It is also interesting to observe that the additionally injected vehicles will clearly represent the majority of the traffic when the number of additional vehicles injected per traffic source is greater than 20.

**Figure 22:**
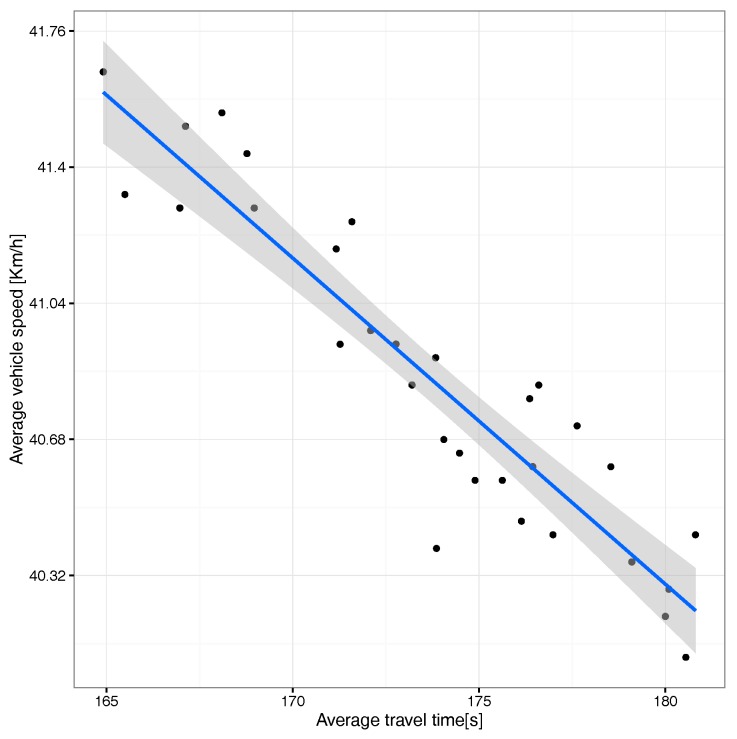
Correlation between average time and average speed.

We now switch our focus to the correlation between average travel time and speed. Figure 22 shows, similarly to what we found before for the uniform scenario, that there is a clear inverse correlation with a thin confidence envelope, although now, the speed variation range is much lower than before (less than 1m/s), meaning that differences are mostly negligible.

**Figure 23:**
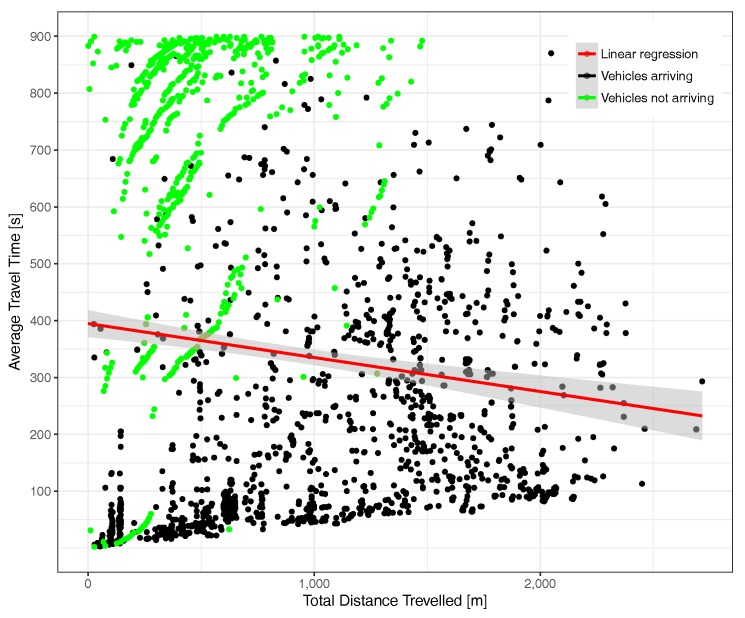
Time-distance correlation under moderate traffic load conditions (total number of vehicles = 1940).

We conclude our analysis by focusing on the relationship between total distance traveled and average travel time. Figure 23 shows the results achieved under low traffic saturation levels. In this case, we can see an unexpected result as the correlation line is negative. This occurs because many vehicles not arriving at their destinations, concentrated near the hotspot area, only travel for a short distance, thereby resulting in this bias. Overall, we can see that the distribution of vehicles arriving at their destination ranges from low to high distances, and from short to long travel times, while vehicles not arriving are concentrated near the upper edge, meaning that they were unable to reach their destination despite being present in the system from the beginning of the experiment in most cases.

**Figure 24:**
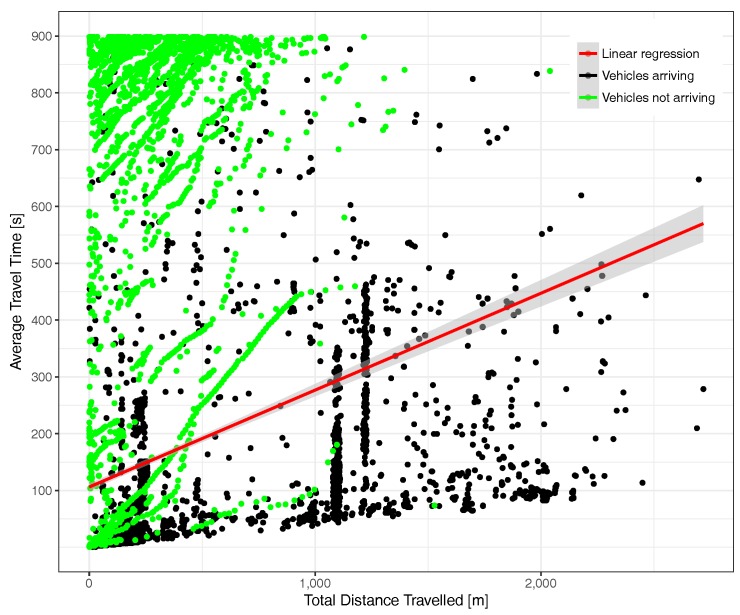
Time-distance correlation under traffic saturation conditions (total number of vehicles = 9985).

Figure 24 shows the same correlation, now under high traffic load conditions. Compared to the previous figure, we can notice two main issues: (i) the correlation between total distance and total time is again proportional and (ii) the vehicular density is higher, and mostly concentrated near the Y axis, similarly to the results obtained for the uniform traffic load scenario.

Overall, the results obtained confirm that the proposed approach allows: (i) seamlessly regulating the amount of traffic in the network, in this case departing from a specific hotspot, (ii) analyzing the impact imposed on the overall traffic system and (iii) proposing alternatives to make traffic flow more efficient even in such extreme congestion situations.

## 7. Conclusions and Future Work

Simulations are a key tool to analyze and improve traffic in our cities. However, for simulation results to be representative, the traffic flows injected must be realistic, meaning that traffic patterns, defined by an origin/destination (O-D) matrix for the vehicles in a city, should be provided.

Valencia is one of the many cities where traffic analysis is of utmost importance, but that nevertheless has no detailed O-D matrix for traffic analysis; in fact, only induction loop measurements for the most relevant streets/avenues are available.

In this paper, we proposed a heuristic that, by iteratively refining the output provided by the DFROUTER tool, is able to generate an O-D matrix for the traffic in Valencia that attempts to be a good approximation of the real traffic distribution. By comparing the generated results against existing traffic mobility data for the cities of Cologne (Germany) and Bologna (Italy), we found that the traffic flow definitions obtained for Valencia provide realistic results. In particular, we observed a good traffic dispersion throughout the different streets of the city, meaning that traffic is flowing through a high number of street segments. In addition, we found that there was a clear asymmetry between streets/segments with low and with high traffic levels, as occurs in real situations. Moreover, it can be observed that injecting additional vehicles into the road network gradually increases the travel time and decreases the average speed of the vehicles, showing a mostly linear behavior that allows easily testing under different traffic loads.

In general, the results achieved allowed us to be satisfied with the generated O-D matrix and enabled making an analysis of possible traffic optimizations during peak hours for improving travel times and reducing CO${}_{2}$ emissions, which is a topic that remains as future work.

## Figures and Tables

**Table 1:** Iterative heuristic applied in the simulated traffic flow of Valencia city.

*n*	α	$\beta_{n}$	ε	$\varphi_{\min}$	$\varphi_{\max}$	$\varphi_{n}$
0	-	-	-	0	1	1
1	409,499	978,339	1.38926	0	1	0.41857
2	409,499	439,738	0.07384	0	0.41857	0.20928
3	409,499	219,071	−0.46503	0.20938	0.41857	0.31393
4	409,499	329,609	−0.19509	0.31393	0.41857	0.36625
5	409,499	385,108	−0.05956	0.36625	0.41857	0.39241
6	409,499	413,368	0.00945	0.36625	0.39241	0.37933
7	409,499	399,120	−0.02535	0.37933	0.39241	0.38587
8	409,499	406,074	−0.00836	0.38587	0.39241	0.38914
9	409,499	409,153	−0.00084	-	-	-

**Table 2:** General statistic for the three target cities.

City	Area (km${}^{2}$)	# Street Segments	# Segment per km${}^{2}$	Vehicle Density (per km${}^{2}$)
Valencia	77.43	11,418	147.46	173.886991
Cologne	595.9	21,953	36.84	2.31794261
Bologna	2.34	337	144.02	3,751.71

**Table 3:** Statistics about traffic sources in the cities under study.

City	# Source Positions per km${}^{2}$	% of Street Segments Used
Valencia	3.36	2.28
Cologne	1.82	4.71
Bologna	5.13	3.56

**Table 4:** Statistics about traffic destinations in the cities under study.

City	# Destinations per km${}^{2}$	% of Street Segments Used
Valencia	17.13	11.61
Cologne	1.74	4.71
Bologna	4.70	3.26

**Table 5:** Statistics about traffic dispersion in the cities under study.

City	# Occupied Street Segments per km${}^{2}$	% of Street Segments Used
Valencia	95.08	64.48
Cologne	12.88	34.97
Bologna	64.96	45.10
